# Using School-Based Health Programs to Prevent Human Trafficking: The Mount Sinai Experience

**DOI:** 10.5334/aogh.3049

**Published:** 2021-06-08

**Authors:** Angela Diaz, Martha Arden, Silvia Blaustein, Anne Nucci-Sack, Leslie Sanders, John Steever

**Affiliations:** 1 Icahn School of Medicine at Mount Sinai, US

## Abstract

This article describes how school-based health centers can serve as human trafficking prevention sites.

**Setting:**

School-based health centers are available to all students attending a school and are often located in schools whose students have risk factors associated with human trafficking: those with a history of running away from home; unstable housing or homelessness; a history of childhood maltreatment or substance use; LGBTQ-identification; physical or developmental disabilities, including students who have Individualized Education Programs and need special education; gang involvement; and/or a history of involvement in child welfare or the juvenile justice system. The Mount Sinai Adolescent Health Center provides a model of the types of service school clinics can offer, including integrated medical, sexual, and reproductive health, health education, and behavioral and mental health.

**Activities:**

Identifying young people with risk factors and addressing those factors in our clinics in a timely way can disrupt the progression to human trafficking. In addition, if young people who are trafficked are attending schools that have a clinic, their health needs, such as care for sexually transmitted infections and mental health issues, can be addressed on-site. Lastly, some people go to school to recruit students for human trafficking. By raising awareness and addressing human trafficking in the school, students can become aware of this issue and perhaps gain the ability to ask for help if they are approached or know of other students being recruited by a trafficker.

**Implications:**

The location of easily-accessible, adolescent-friendly, trafficking-aware services in schools can prevent, identify and intervene in human trafficking.

## Program Description

School-based health centers (SBHCs) are clinics that are located within school buildings to increase the accessibility of medical and mental health services to young people. The model, which has expanded widely in the United States over the past 50 years, generally employs an advanced practice provider or physician and a social worker or other mental health provider, in addition to a variety of support staff, to assess, diagnose, and treat medical and mental health conditions within schools so young people do not have to leave school for medical appointments. Nationally, there were over 2,300 traditional school-based health centers in the United States during the 2016–2017 school year, with close to half located in urban schools and about 35% in rural areas. The majority are located in the 17 states that directly provide SBHC funding, half operated by Federally Qualified Health Centers and about a quarter operated by hospital systems [1].

By eliminating many of the barriers to care and serving adolescents where they are most independent, the SBHC model provides a unique opportunity to prevent human trafficking. Mount Sinai Adolescent Health Center (MSAHC), founded in 1968, opened its first school-based health center (SBHC) in 1983, in response to the overwhelming needs of the East Harlem community of New York City, where one neighborhood high school had a graduation rate of about 5% for students entering ninth grade. The locally-controlled school district was eager to institute major changes to transform the failing school, and incorporated the first SBHC in New York City as part of that transformation. With the support of the New York State and New York City Departments of Health the program grew, and MSAHC now operates SBHCs inside six Manhattan school buildings enrolling over 11,000 students. The SBHCs are staffed by a dedicated team of 27 physicians, nurse practitioners, social workers, health educators, and medical assistants who work together to promote wellness and provide health education and integrated, comprehensive health care. The SBHC services include health promotion, health maintenance, health screenings, diagnosis and treatment of acute illness and injury, management of chronic disease, sexual and reproductive health services, mental health crisis intervention, and individual and group psychotherapy.

The program is funded by state and city Department of Health grants, Medicaid billing, in-kind contributions from MSAHC of staff and resources, and philanthropy; there are never any out-of-pockets costs for users, regardless of insurance or immigration status. The annual budget for the Mount Sinai program averages about $600,000 per site. Entitlement coordinators at the MSAHC actively assist young people and their families in obtaining insurance coverage, providing them with increased access to additional sources of care and enabling the program to generate much-needed income for services rendered at the SBHCs.

## Program Activities

### SBHC Model

School-Based Health Centers are comprehensive, interdisciplinary, integrated health care clinics located within school buildings, generally available only to the students attending that school. The SBHC model has been developed to provide accessible, convenient, and teen-friendly health care services in a familiar environment, removing many of the barriers to care that are often faced by young people and, as such, providing a unique opportunity to prevent a number of conditions, including human trafficking. SBHCs are available to every student in the school building, and there is never any charge to the students for services. SBHC services are available when and where the teens are present, and parents do not need to take time off from work to attend children’s health visits. Parental consent for participation is obtained, whenever possible, when a student first enrolls in the school, and adolescents are seen on an appointment or walk-in basis without a requirement for parental notification. Because SBHCs provide a wide variety of services, including routine physical exams and immunizations as well as visits for more sensitive reasons, SBHC use is not stigmatized. Confidentiality is strictly protected, and all services use a single waiting room so that the reason for visit is not apparent to peers.

In addition to removing many barriers to obtaining care, the teen-centered atmosphere and the ease of keeping confidentiality make SBHC care especially attractive to young people, and several studies have demonstrated that students preferentially use SBHC services [2]. Students at schools with SBHCs report greater awareness of the availability of confidential services and higher rates of discussion of sexual activity, contraception, and emotions, lending further support to the model [3]. School-based health centers reach the high-need students in their buildings. Compared with students who do not use health centers in their school, SBHC users are more likely to be involved in risky health behaviors, report exposure to violence and drug culture, and have a history of suicidal ideation or attempts, school suspension for fighting, and school failure [4]. For all these reasons, SBHCs are ideally positioned to help prevent human trafficking, and the SBHC’s relationships with school personnel afford the additional opportunity to increase awareness of human trafficking through classroom presentations.

### MSAHC SBHC Activities and Staff

The Mount Sinai School-Based Health Centers provide integrated physical health, sexual and reproductive health, health education, and behavioral and mental health services in offices that are located within the school buildings but operate independently (and collaboratively) with the school administration and Department of Education. Certified medical assistants serve as the welcoming face of the SBHCs, greeting and registering patients, calming nervous and upset teens, interacting with school personnel and parents, and managing the flow of the clinic. Health educators play a key role in educating patients about healthy lifestyle, positive decision making, and pregnancy and sexually transmitted infection (STI) prevention. Young people learn about the various types of contraceptives and how to use protection to prevent STIs. The health educators also provide HIV pre-test counseling, HIV testing, and HIV post-test counseling as well as engaging patients in discussions of abstinence and safe sex, providing them with support and skills for making healthy decisions.

Using a patient-centered approach that recognizes the critical role of psychosocial issues and behaviors in the medical care of teens, physicians and nurse practitioners take extensive health histories and provide comprehensive physical examinations, immunizations, health and nutrition education, and laboratory tests. Students are routinely asked about sexual orientation and gender identity, and sexual activity is discussed at all medical visits. Screening for sexually transmitted infections and HIV testing is encouraged, with appropriate reproductive health anticipatory guidance provided for both sexually active and abstinent patients. Condoms are freely available to all patients, and all FDA-approved methods of contraception—including the pill, patch, ring, injection, and long acting reversible contraceptives (LARCs) including IUD and Nexplanon—as well as emergency contraception, are available on-site.

At each medical visit, emotional well-being is assessed, including psychosocial history and use of screenings for substance use, exposure to interpersonal violence, and depression using a PHQ-2 screening tool [5]. The medical history also includes a determination of the strengths and areas of potential challenges, such as having a history of childhood maltreatment, involvement in foster care or the juvenile justice system, of running away, having an individualized education plan (special education), or of having an unstable living situation or being homeless. Students found to have any behavioral health or substance use concens are connected to the social worker in the program for assessment and appropriate treatment.

Licensed clinical social workers work in an integrated model, closely collaborating with the medical personnel at their site. They see patients for immediate evaluation as part of their primary care visit if the medical provider identifies a need, but also provide ongoing psychotherapy, incorporating principles of Cognitive Behavioral Therapy (CBT), Dialectical Behavioral Therapy (DBT), and other evidence-based techniques when indicated. Patients are connected to the social workers by other members of the school-based health center team, including the medical providers, medical assistants, and health educators, but school personnel, parents, and students themselves are also important sources of referrals.

### Program Outcomes and Evaluation Metrics

The MSAHC’s SBHCs program serves five thousand students in one academic year, who make over 20,000 visits per year. Of those visits, about one quarter of the visits are for sexual and reproductive health services, and about a quarter of the visits are for mental health services.

The program tracks and analyzes a number of quality indicators each year. In each site, over 75% of students enrolled in the school buildings served by the SBHC program are enrolled in the SBHC. Over 98% of students seen at each site were screened for sexual activity, with 48% acknowledging sexual activity. Thousands of condoms were distributed at each site, and all sexually active females were offered contraceptive methods, including LARCs. Fifty-five percent of sexually active females received more effective contraception (pill, patch, ring, or injection), and another 13% elected to use LARC (IUD or Nexplanon). Of those engaging in sexual activity, over 80% were screened for chlamydia within the preceding year, and 100% who tested positive were treated program-wide. Virtually all students (97–99.7%) seen for medical visits at the six sites were screened for depression, and over 85% of those with a positive screen were seen by the SBHC social workers (others declined referral). A small percentage of our patients acknowledge involuntary sexual activity; all of them were referred to our SBHC social workers for evaluation and counseling, and all were offered sexually transmitted infection screening and contraception. Screening for obesity, asthma treatment, and immunization completion are also tracked.

## Reflections

### Program Strengths

The major strength of SBHCs is accessibility: students come into the health center, are evaluated and treated, and then return to class. This reduces time away from the classroom due to outside appointments or illness, and greatly decreases barriers to care faced by teens. The immediate on-site availability of services for urgent medical situations, such as asthma attacks, is especially helpful for students and their families. In addition, the school-based model lends itself to close follow-up and continuity of care. Reminders are sent within the school to students who miss apointments at the SBHC, and they can be retrieved from the classroom when necessary.

Another major strength of the MSAHC’s school-based health program is that the teen-friendly staff teaches students how to access and utilize health care services in a supportive environment, supporting them through adolescence and preparing them for the responsibilities of adulthood. This is a unique position to identify risky bevariors, including a history or risk of human trafficking.

### Program Challenges

The biggest challenge, unsurprisingly, is funding. With 1,500 to 2,500 students in each school building, there is enough need to keep several physicians, social workers, medical assistants, and health educators busy, but grant funding and Medicaid income are inadequate to cover the costs of even the bare minimum level of staffing. The MSAHC is very active in grant-seeking activities and philanthropy to help fund the SBHCs and special initiatives and programs. Space is also a major issue, and although the Department of Education has been generous in allowing the SBHCs to use some of their precious space, the program was unable to expand one site even when funding for construction and additional staff had been secured because the school did not have the additional space to give for the clinic expansion.

### SBHCs and Human Trafficking

The features of SBHCs offer a unique opportunity to prevent and interrupt human trafficking: these clinics are well poised to prevent human trafficking because they serve students with conditions known to be risk factors for human trafficking [6]. When these risk factors are identified, they can be addressed in a timely way in an effort to prevent or interrupt the progression to human trafficking. These risk factors include having a history of childhood abuse [7,8]; a history of systems involvement in child welfare, including foster care [8]; having unstable living situations, being homeless, or having a history of running away [9]; use of alcohol and other drugs [10]; a history of mental health needs, including suicidality [7,11]; or identifying as LGBTQ [8,12]. All of these risk factors are assessed in the SBHC, and the mental health staff play a key role in providing not only counseling, but also assisting with referrals for concrete services. An affiliation with two legal services organizations, Youth Represent and New York Legal Aid, provide additional resources for program participants who have housing needs, and a trangender care program at MSAHC provides specialized services and group support for these at-risk teens.

The staff of the SBHC can bring awareness about the issue of human trafficking both in one-on-one interactions with students in the clinics and by doing presentations in the classrooms. By developing relationships with the students and providing a safe space where students feel comfortable, students are able to come for guidance and raise issues of concern, such as being approached by people outside the school, intimate partners, family members, gangs or others with activities that appear to be consistent with human trafficking. We also need to be aware that sometimes recruitment into trafficking occurs in school [6]. This fact can be incorporated in the work with students and school personnel. Training of school personnel on the issue of human trafficking will bring awareness to the staff in the school, and perhaps because of this they will be more likely to see signs or behaviors that may indicate involvement in human trafficking.

Staff members at the school-based health centers who serve these students identify these risk factors and address them in mental health, health education, and medical services. By identifying and serving these young people and providing the treatment they need in a trauma-informed and trauma-responsive environment, the SBHC can provide or connect students to trauma-specific interventions, which help young people heal and become less likely to end up in a trafficking situation. Students with a history of victimization have increased utilization of both mental health and medical services [13,14], and the convenience of school-based services can further increase the engagement of these youth.

School-based health centers can also provide primary prevention by doing anticipatory guidance during routine medical visits, helping young people be aware of human trafficking and the concept of grooming. Knowing that the school-based health center is a resource for them if they ever have a question or are not sure of a situation is critically important: they can always go to the school clinics and talk to the staff.

### Lessons Learned and Implications for Other Programs

School-based health services need to be extremely flexible and accessible. Demand for services in school is based on the students’ schedules, and health center staff members have to be ready to see patients during the times when students have breaks, if possible. They also must be ready to complete visits comprehensively but quickly when the student has a test or other school obligation. SBHCs’ staff are aware and understand that many students are unable or unwilling to stay after school for an appointment, even when the appointment is for a service they want and need.

Another lesson is that it is important to have a continuum of care available because schools are frequently closed for holidays and vacations—and even if school buildings are open during vacations, students are unlikely to use school-based health services when school is not in session. During those times, the community-based MSAHC can provide continuity of care to all its school-based health patients because the SBHCs use the same electronic medical record system and familiar providers from the school-based health staff work at MSAHC during the summers when students are on vacations. The students know that if they ever need services when the schools are closed, they can get those services at MSAHC because the SBHCs are integrated with MSAHC. It is really one integrated program with clinics in schools and in the community. In addition, since school-based health services are not available to teens after graduation, it is essential to have a strong linkage to ongoing care, as is provided by the MSAHC to youth up to age 26.
